# 

**Published:** 1920-06

**Authors:** 


**The Library is now open daily from 9 a.m. to 7 p.m. continuously, except on Saturdays, when it closes at 1 p.m.**

**The Library is closed during August.**

*The following donations have been received since the publication of the list in March.*

*June* 15*th*, 1920.

| | | |
|---|---|---|
| American Medical Association (1) | .. .. | 1 volume. |
| Carey Coombs, M.D. (2) .. | .. .. .. | 1 ,, |
| D. S. Davies, M.D. (3) .. | .. .. .. | 2 volumes. |
| J. M. Fortescue-Brickdale, M.D. (4) | .. .. | 3 ,, |
| E. W. Hey Groves, M.D. (5) | .. .. .. | 8 ,, |
| The Medical Research Council (6) | .. .. | 1 volume. |
| E. Watson-Williams, M.B. (7) | .. .. .. | 1 ,, |

### THE ONE HUNDREDTH LIST OF BOOKS.

The figures in brackets refer to the figures after the names of the donors, and show by whom the volumes were presented. The books to which no such figures are attached have either been bought from the Library Fund or received through the *Journal*.

| | | |
|---|---|---|
| **Albee, F. H.** | *Orthopedic and Reconstructional Surgery* | 1919 |
| **Austen, E. E.** | *The House Fly : Life History, etc.* | 1920 |
| **Barton, G. A. H.** | *Backwaters of Lethe* | 1920 |
| **Brown, C. F. White and W. H.** | *An Atlas of Syphilis* | 1920 |
| **Brown, W. L.** | *The Sympathetic Nervous System in Disease* | 1920 |
| **Cabot, R. C.** | *Differential Diagnosis.* Vol. I. .. 4th Ed. | 1919 |
| | *Cerebro-Spinal Fever among the Military Forces,* 1915–1919 .. (6) | 1920 |
| **Collis, E. L.** (Ed.) | *The Industrial Clinic* | 1920 |
| **Cruchet and R. Moulinier, R.** | *Air Sickness* (Trans. by J. R. Earp) | 1920 |
| **Cunningham, D. J.** | *Manual of Practical Anatomy* (Ed. by A. Robinson), Vols. II. and III. .. 7th Ed. | 1920 |
| **Fletcher, and H. McLean, C.** | *The Link between the Practitioner and the Laboratory* | 1920 |
| **Friel, A. R.** | *Electric Ionization* | 1920 |
| **Gray, H.** | *Anatomy* (Ed. by R. Howden) .. 21st Ed. | 1920 |
| **Green, T. H.** | *Pathology and Morbid Anatomy* (Ed. by H. M. Murray) .. 9th Ed. | 1900 |
| **Harrison, L. W.** | *A Manual of Venereal Disease for Students* | 1920 |
| **Hart, A. H.** | *The Doctor's Manual* .. 4th Ed. | 1920 |

**Holt and J. Howland, L. E.** *Diseases of Infancy and Childhood* 7th Ed. 1919
**Howland, L. E. Holt and J.** *Diseases of Infancy and Childhood* 7th Ed. 1919
**James, S. P.** .. .. *Malaria at Home and Abroad* .. .. .. .. .. 1920
**Kenwood, H. R.** .. *Public Health Laboratory Work* (*Chemistry*) 7th Ed. 1920
**Lang, W. D.** .. .. *British Mosquitoes* .. .. .. .. .. .. .. 1920
*Malaria in England in* 1917, *Reports on* (L.G.B.) .. .. .. (4) 1918
**Masters, W. E.** .. *Essentials of Tropical Medicine* .. .. .. 1920
**McLean, C. Fletcher and H.** *The Link between the Practitioner and the Laboratory*.. .. .. .. .. .. .. .. .. 1920
**Miles, A. Thomson and A.** *Operative Surgery* .. .. .. 3rd Ed. 1920
**Miller, H. E.** (Ed.) *Functional Nerve Disease* .. .. .. .. 1920
**Morris, Sir M.** .. *The Story of English Public Health* .. (3) [1919]
**Moulinier, R. Cruchet and R.** *Air Sickness* (Trans. by J. R. Earp).. 1920
**Owen, R.** .. .. *Lectures on Comparative Anatomy.* 2 vols. 1843–46
**Paton, D. N.** .. .. *Essentials of Human Physiology* .. 5th Ed. 1920
**Quain, R.** .. .. *Clinical Lectures* .. .. .. .. .. .. .. 1884
**Saberton, C.** .. .. *Diathermy in Medical and Surgical Practice*.. 1920
**Schuster, E.** .. .. *Eugenics* .. .. .. .. .. .. .. .. .. (3) [1912]
**Short, A. R.** .. .. *The New Physiology in Surgical and General Practice* .. .. .. .. .. .. .. 4th Ed. 1920
**Stewart, Sir J. P.** .. *The Diagnosis of Nervous Diseases*.. 5th Ed. 1920
**Sunde, A.** .. .. *Chorioepithelioma Malignum* .. .. .. .. 1920
**Thomson and A. Miles, A.** *Operative Surgery* .. .. .. 3rd Ed. 1920
**Walsham, W. J.** .. *Surgery* .. .. .. .. .. .. .. .. 8th Ed. 1903
**Watson-Williams, E.** *Selenium in Cancer* (T.S.) .. .. .. (7) [1920]
**Webb, S. and B.** .. *The State and the Doctor* .. .. .. .. .. .. 1910
**White and W. H. Brown, C. F.** *An Atlas of Syphilis* .. .. .. 1920

### TRANSACTIONS, REPORTS, JOURNALS, ETC.

American Journal of the Medical Sciences, The (4) Vols. CLVI.—CLVII. 1918–19
American Medical Association, Transactions of the Section on Genito-Urinary Disease .. .. .. .. .. .. .. .. .. .. .. .. .. (1) 1916
American Ophthalmological Society, Transactions of the Vol. XVII. 1919
British Medical Journal, The .. .. .. .. .. .. Vol. II. for 1919
College of Physicians of Philadelphia, Transactions of the Vol. XL. 1918
English Catalogue of Books .. .. .. .. .. .. 1914, 1915 ; 1919, 1920
International Abstract of Surgery.. (5) Vols. XXVI.—XXIX. 1918–19
Lancet, The .. .. .. .. .. .. .. .. .. .. .. .. Vol. II. for 1919
Medical Annual, The .. .. .. .. .. .. .. .. .. .. .. .. .. 1920
Quarterly Journal of Medicine .. .. .. .. .. (2) Vol. XII. 1918–19
Royal Academy of Medicine in Ireland, Transactions of the Vols. XXXVI.—XXXVII. 1920
Surgery, Gynecology and Obstetrics (5) Vols. XXVI.—XXIX. 1918–19

**University of Bristol.**—Students of the University have recently passed the following examinations :—

University of Bristol Diploma of Dental Surgery.—*Third Examination :* N. H. Bodenham, K. G. Hyland.

F.R.C.S. Eng.—*Primary Examination :* A. W. Fawcett, J. W. Gilbert, E. Watson-Williams.

M.B., B.S. Lond.—*Third Examination :* J. A. Birrell.

Conjoint Examining Board of England.—*Medicine :* M. A. Distaso, H. V. Jackson. *Midwifery :* H. V. Jackson.

L.D.S., R.C.S. Eng.—*Final Examination :* W. G. Milton, L. H. Buchan.

